# Premalignant Conditions of Bone

**DOI:** 10.5435/JAAOSGlobal-D-22-00097

**Published:** 2022-10-13

**Authors:** Michael D. Eckhoff, Matthew E. Wells, Osvaldo Padilla, Elizabeth M. Polfer, Christopher J. Castagno, Ahmed M. Thabet, Shaimaa Elzamly, Harry L. Wilson, Rajiv Rajani

**Affiliations:** From the Department of Orthopedics, Texas Tech University Health Science Center El Paso, El Paso, TX (Dr. Eckhoff, Dr. Wells, Dr. Thabet, and Dr. Rajani), Department of Orthopedics, William Beaumont Army Medical Center, Ft. Bliss, TX (Dr. Eckhoff, Dr. Wells, and Dr. Polfer), Department of Pathology, Texas Tech University Health Science Center El Paso, El Paso, TX (Dr. Padilla and Dr. Wilson), Paul L. Foster School of Medicine, Texas Tech University Health Science Center El Paso, El Paso, TX (Mr. Castagno), and the Department of Pathology, University of Texas Health Science Center at Houston, Houston, TX (Dr. Elzamly).

## Abstract

Development of malignancy is a multifactorial process, and there are multitude of conditions of bone that may predispose patients to malignancy. Etiologies of malignancy include benign osseous conditions, genetic predisposition, and extrinsic conditions. New-onset pain or growth in a previously stable lesion is that should concern for malignant change and should prompt a diagnostic workup for malignancy.

Malignant conditions of bone can arise from numerous sources, and the exact etiology is not always known. Early identification of malignancy equates to earlier appropriate treatment and improved long-term patient outcomes.^1^ Several conditions have been associated with a higher risk of malignant transformation. These conditions include benign osseous lesions with delayed malignant transformation, genetic predispositions to malignant degeneration, and extrinsic influences. This review discusses a multitude of diagnoses and factors that fall into these three categories.

Malignant transformation often results from abnormalities in either tumor suppressor genes or proto-oncogenes. Tumor suppressor genes are normal genes that regulate cellular processes, such as cell division, DNA repair, and apoptosis. When these genes are mutated, the normal regulation of these processes is lost and cells are able to proliferate and survive in an uncontrolled manner. Examples include *TP53* and *RB*.^2,3^ Inherited disorders, such as Li-Fraumeni and Retinoblastoma, associated with tumor suppressor genes often affect one of the two copies of the gene. The patient subsequently develops a mutation in the second copy of the gene, causing malignant transformation.^4^ Proto-oncogenes are normal genes that promote cellular growth and proliferation, which can become constitutively active due to gene mutation, examples being *RET* and *BCL-2*.^5,6^ Activation of proto-oncogenes results in unchecked growth and proliferation of cells, which can lead to papillary thyroid carcinoma,^7^ osteosarcoma,^8^ and lymphoma.^9^ Often tumor suppressor gene mutations are inherited, while proto-oncogene mutations are acquired.^10^ Table 1 presents all conditions included in this review and their associated protein mutations.

**Table 1:** Premalignant Conditions, Associated Mutated Protein, and Protein Function

Condition	Associated Mutated Protein (Gene)	Protein Function
Osteochondroma	Exostosin-1,2,3 (*EXT1, EXT2, EXT3*)	Heparan sulfate biosynthesis
Enchondroma	Isocitrate dehydrogenase-1&2 (*IDH1&2*)	Tricarboxylic acid cycle
Paget disease of bone	Sequestosome-1 (*SQSTM1*)	Autophagosome cargo protein
Fibrous dysplasia	G-protein (*GNAS*)	Signal transduction protein
Synovial chondromatosis	No associated gene mutation	
Chondroblastoma	H3.3 histone B (*H3F3B*)	Nucleosome structure and genetic integrity
Giant cell tumor of bone	H3.3 histone A (*H3F3A*)	Nucleosome structure and genetic integrity
Osteoblastoma	c-Fos (*cFOS*)	Proto-oncogene, target gene promotor and enhancer
Retinoblastoma	Retinoblastoma (*RB1*)	Tumor suppressor gene, cell-cycle checkpoint regulation
Li-Fraumeni	Tumor protein p53 (*TP53*)	Tumor suppressor gene, cell-cycle checkpoint regulation
Rothmund-Thompson	RecQ helicase (*RECQL4*)	Telomerase maintenance protein
Bloom syndrome	RecQ helicase (*BLM*)	Telomerase maintenance protein
Werner syndrome	RecQ helicase (*WRN*)	Telomerase maintenance protein
Osteomyelitis	No associated gene mutation	
Postradiation sarcoma	Cyclin dependent kinase inhibitor 2A&B (*CDKN2A&B*)	Tumor suppressor gene, cell-cycle checkpoint regulation

## Benign Osseous Conditions With Delayed Malignant Transformation

### Osteochondroma

**Figure 1:**
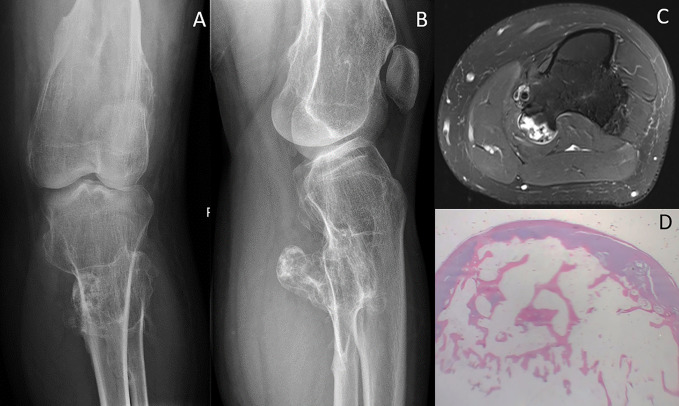
Diagrams showing multiple hereditary exostoses: (**A** and **B**) demonstrate AP and lateral radiographs with characteristic exostoses with secondary deformity of the knee joint. **C**, Axial T2 MRI cut with signal intense cartilaginous cap. **D**, Complete resection of this lesion shows a cartilaginous cap with underlying trabecular bone and marrow contents.

Solitary osteochondromas are the most common benign lesions of bone accounting for approximately 30% of all benign bone lesions (Table 2).^11^ These are often referred to as exostoses and can arise in any bone of the body, but most often develop in areas of notable growth, such as the distal femur, proximal tibia, and proximal humerus. The true incidence of osteochondromas is likely underrepresented because they often present as incidental findings on imaging studies.^12^ These exostoses are characterized as pedunculated or sessile boney masses in direct continuity of the medullary canal with an overlying cartilage cap^13^ (Figure 1, A–C). Histologically, these “mushroom” shaped lesions show a cartilaginous cap composed of mature hyaline cartilage with normal underlying bone that includes trabecular bone and marrow contents (Figure 1D). Malignant transformation can occur within the cartilage cap with degeneration to chondrosarcoma and occurs in approximately 1% of benign cases.^14,15^ The exception to this low rate of malignant transformation in osteochondromas is found in patients with multiple hereditary exostoses (MHE). These patients have a notably increased whole-body risk of osteochondroma degeneration to chondrosarcoma with references rates as high as 35%.^16,17^ More recent MHE studies, however, suggest much lower rates of malignant transformation at around 2% to 5%.^13,18,19,20,21^

**Table 2:** Summary of Key Points

No	Benign Osseous Conditions
1	Osteochondromas and enchondromas are benign cartilaginous lesions of bone with low rates of malignant degeneration which is increased in conditions characterized by multiple lesions
2	Paget disease of bone and fibrous dysplasia are disorders of disorganized or dysplastic bone formation and can develop osteosarcoma secondarily
3	Synovial chondromatosis is a metaplastic process with low rates of chondrosarcomagenesis
4	Chondroblastoma and giant cell tumor of bone are benign bone tumors associated with lung metastases and rarely develop malignant transformation posttreatment of an initial lesion

MHE is an autosomal dominant genetic predisposition to the development of multiple diffuse osteochondromas.^22,23^ Mutations in the *EXT1*, *EXT2*, and *EXT3* genes have been attributed to this condition.^24^ All three *EXT* gene proteins function in heparin sulfate proteoglycan biosynthesis with loss of function mutations resulting in dysregulated growth.^25^ While occurring almost equally between male and female patients, the more severe phenotype predominates in male patients.^18^ Common symptomatology among these patients includes localized nerve compression, limb-length discrepancies, and genu valgum.^26^

Malignant transformation of an osteochondroma is associated with several symptoms. Previously dormant lesions that insidiously continue growing, particularly after skeletal maturity, can be suggestive of malignant transformation.^27^ This is especially true if the lesion becomes painful without a clear etiology.^27^ In addition, osteochondromas with a cartilage cap greater than 2 cm by radiographic imaging are associated with the development of chondrosarcoma.^28^ This cartilage cap may be measured using MRI or CT scan images with higher interobserver reliability for measurements obtained on CT imaging.^28^

When this malignant transformation does occur, it is most often to low-grade chondrosarcoma that can often be treated effectively with wide excision alone and with a good prognosis at >90% survival.^17,27,29^ Patients with chondrosarcoma arising from osteochondromas in the axial skeleton, particularly in the pelvis, may have worse outcomes due to delays in identification and subsequent treatment.^27,30^

### Enchondroma

**Figure 2:**
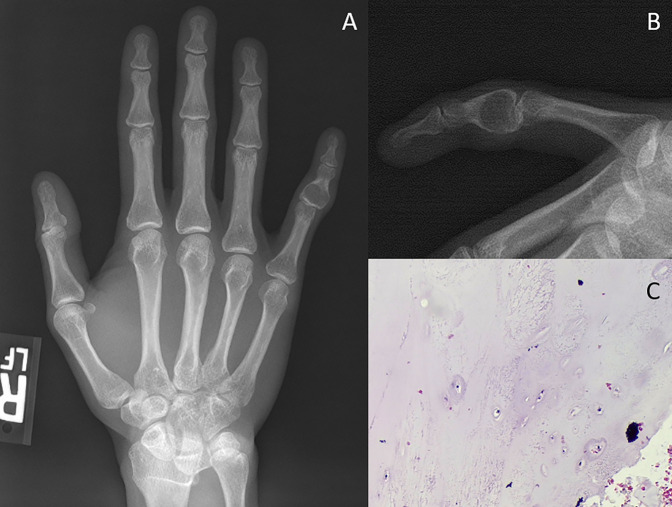
Diagrams showing enchondroma. **A**, AP and (**B**) lateral radiographs of a small finger middle phalanx base enchondroma. Often these lesions are purely lytic and expansile when present in the small bones of the hand. **C**, Hyaline cartilage is seen with variable atypia. In this case, the atypia is minimal, but sometimes the atypia may resemble low-grade chondrosarcoma due to hypercellularity, nuclear atypia, and myxoid changes.

Characterized as benign intramedullary hyaline cartilaginous tumors, enchondromas are one of the most common primary bone tumors in the body^31^ (Figure 2, A and B). They account for 3% of all bone tumors and 13% of all benign bone tumors.^32^ The exact incidence is unknown because these are typically asymptomatic and found incidentally.^33^ As these lesions are benign, treatment is most often with observation alone when found. Tumors that do present, often present secondarily to a pathologic fracture, because they can create a relative area of weakness in the bone.

Malignant transformation is of notable concern in patients who have enchondromatosis.^31,34^ Two main subtypes of enchondromatosis include Ollier disease and Maffucci syndrome. Both disorders are nonhereditary, and the malignant transformation occurs most often during the fourth decade of life.^35^ Isolated enchondromas, Ollier disease, and Maffucci syndrome are all associated with mutations in *IDH1* and *IDH2* genes, encoding proteins involved in the tricarboxylic acid cycle with downstream effects on histone modification and DNA hypermethylation.^36,37^ Rates of malignant transformation are 10% to 40% for Ollier disease and up to 15% to 50% for Maffucci syndrome.^35,38,39^ Other malignancies can occur as well in these patients including astrocytoma, gliomas, and mesenchymal ovarian tumors.^39,40^

Although uncommon, the most common secondary malignancy to occur in the setting of an enchondroma is dedifferentiation into chondrosarcoma. Differentiating a benign enchondroma from low-grade chondrosarcoma can be difficult, both on radiographic evaluation and biopsy analysis.^38,41^ Signs and symptoms of malignant transformation include the development of a mass in the region of a previously known enchondroma or, very importantly, new-onset pain. Radiographic findings include periosteal reaction, endosteal scalloping, soft-tissue invasion, and poorly demarcated lesions.^42^ Microscopically, they show lobules of hyaline cartilage that are often encased by bone or fibrous perichondrium (Figure 2C). Treatment for low-grade chondrosarcomas of the extremities and symptomatic enchondromas is the same and usually entails marginal curettage excision, bone grafting, and/or polymethylmethacrylate augmentation.^38^ Low-grade pelvic chondrosarcomas and all higher-grade chondrosarcomas should be treated with wide resection.^43^ Prognosis is best when enchondromas occur in the short bones of the body.^35^

### Paget Disease of Bone

**Figure 3:**
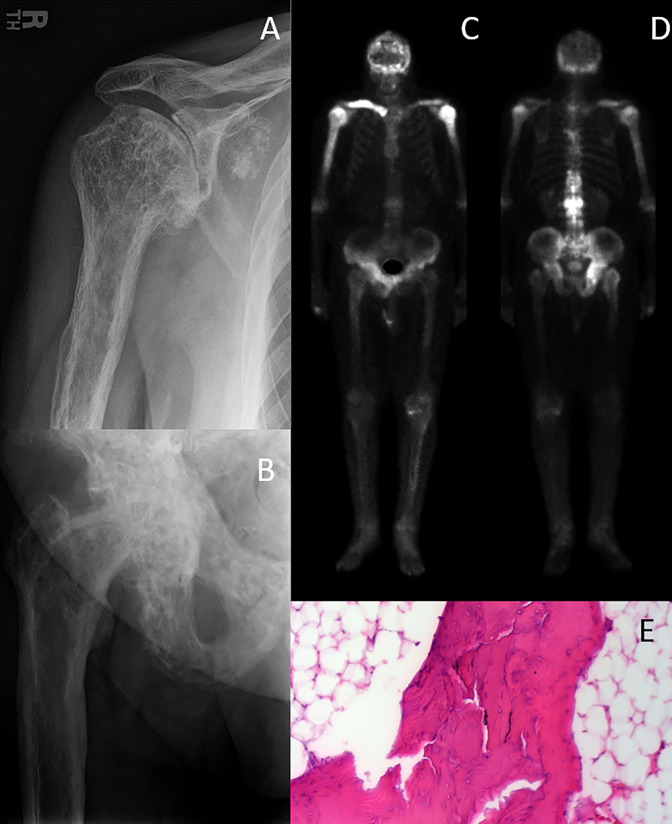
Diagrams showing polyostotic Paget disease of bone. **A**, Proximal humerus and (**B**) proximal femur and pelvic involvement with classic moth-eaten appearance. **B**, Varus deformity can develop secondary to proximal femoral involvement. Technicium-99 bone scan (**C**) anterior and (**D**) posterior showing increased metabolic activity in bilateral proximal humeri, the right clavicle, and right hip. **E**, This microscopic image shows trabecular bone with a mosaic (or jigsaw puzzle) pattern, along prominent cement lines.

Paget disease of bone, also referred to as osteitis deformans, is a metabolic disorder characterized by osteoclast-mediated disorganized bone remodeling,^44^ typically found in patients aged older than 55 years.^45,46^ It most often affects people of European descent with a predilection for the axial skeleton. Although the exact mechanism is not understood, it is thought to be secondary to multiple environmental factors, including nutrition, infection, and activity level of the patients.^47^ Radiographically, it is characterized by osteolytic extension from epiphysis toward metaphysis with a widening of the affected bone with coarsened trabeculae and cortical thickening^48^ (Figure 3A–D). The histology varies based on the temporal phase of this lesion: osteolytic phase, mixed osteoclastic/osteoblastic phase, or osteosclerotic phase. The earlier presentation shows woven bone and a mosaic pattern (or jigsaw puzzle) appearance of lamellar bone along cement lines, (Figure 3E), while the later stages show thick bone trabeculae with myelofibrosis.

A genetic predisposition for Paget disease of bone has been established as displayed through a mutation in the ubiquitin-associated domain of the *SQSTM1* gene; this resulting mutation displays autosomal dominance with variable penetrance.^49^ Paget osteosarcoma, often referred to as Paget sarcoma, is a devastating complication of Paget disease of bone with poor outcomes.^50,51,52,53,54^ Although osteosarcoma is the most common subtype of Paget-associated tumor, chondrosarcoma and fibrosarcoma are also documented.^55^ This malignant transformation thankfully occurs in only approximately 1% to 3% of cases.^56,57^ The rate of transformation is higher in severe polyostotic Paget patients at 5% to 10%.^48^ Symptoms of transformation include acute-onset pain or sudden increase in a previously stable chronic pain. Additional signs can include swelling or the development of a soft-tissue mass. Radiographically, malignancy is characterized by invasive growth within the medullary canal, cortical destruction, and soft-tissue expansion.^57^ The femur, humerus, and skull are most often affected by the sarcomatous transformation.

Diagnosis and treatment, when a malignant transformation is suspected, should be confirmed with a biopsy and followed with early aggressive treatment. Overall prognosis with Paget sarcoma is poor with 80% to 90% of patients dying within 3 years.^48,58,59^ These poor survival rates may be secondary to the frank malignant disease on presentation or simply due to its occurrence in older, more medically comorbid patients who cannot tolerate the aggressive chemotherapeutic and surgical treatment options. Treatment can include surgery, chemotherapy, and radiation therapy and is largely dependent on the sarcoma that develops.^60^ Fortunately, the rates of Paget sarcoma seem to be declining overall.^58^

### Fibrous Dysplasia

**Figure 4:**
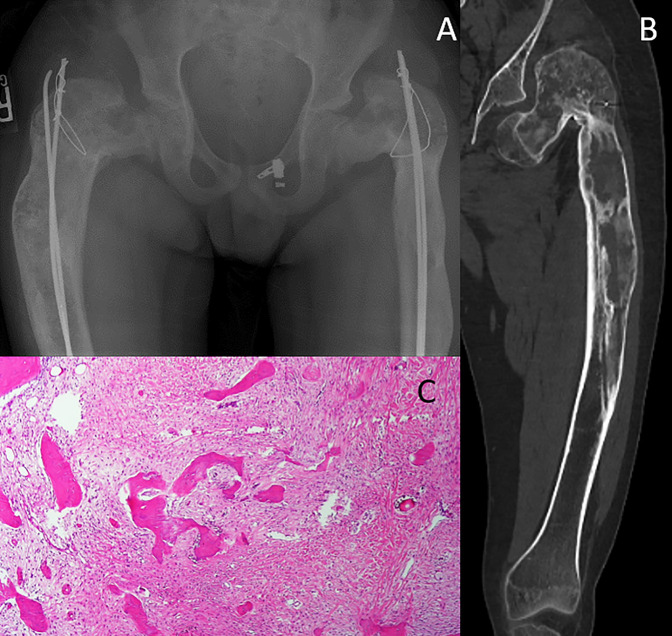
Diagrams showing polyostotic fibrous dysplasia. **A**, AP pelvis x-ray demonstrating bilateral expansile and ground-glass appearing lesions with secondary varus deformity of the proximal femur. **B**, CT of the patient's left femur after hardware removal with Sheppard crook deformity. **C**, These lesions typically show irregular shaped, thin bone trabeculae in a background of fibroblast-like spindle cells, which lack conspicuous osteoblastic rimming.

As a relatively common lesion, fibrous dysplasia (FD) has been well described in the literature. Occurring as both mono-ostotic and polyostotic, FD is a disorder where fibro-osseous bone forms in lieu of native bone marrow with cancellous bone. The etiology for FD is a *GNAS* gene mutation with downstream constitutive activation of cAMP production and activation of the parathyroid hormone receptor.^61^ Monostotic FD comprises 75% of cases,^62^ often presenting in the second to fourth decades of life secondary to pain or pathologic fracture. Polyostotic patients often present earlier and are more likely to have an associated limb deformity present. On radiographic examination, the affected bone has a classically coined ground glass appearance (Figure 4, A and B). Histologically, the lesion shows thin trabecular bone in a background of fibroblast-like spindle cells (Figure 4C). These irregular-shaped trabeculae typically lack conspicuous osteoblastic rimming. These osseous locations most commonly affected in descending order include the femur, tibia, pelvis, foot, and facial bones.^63^

There are two major associated genetic disorders with FD, which include McCune-Albright and Mazabraud syndrome. First described by Albright and colleagues, McCune-Albright is known to display a classic triad of polyostotic FD, café-au-lait spots, and precocious puberty, although only roughly half of patients will phenotypically display the triad.^64^ Mazabraud syndrome is polyostotic FD with intramuscular myxomas. Similar to Paget disease, FD most commonly undergoes malignant transformation to osteosarcoma, chondrosarcoma, and fibrosarcoma at a rate of approximately 1%.^65^ However, malignant transformation is more common with polyostotic involvement with rates of around 4% in both McCune-Albright and Mazabraud patients.^61,66^ The most common locations for malignant transformation are, unsurprisingly, the proximal femur, humerus, and pelvis. Historically, one of the contributing factors to malignancy has been the treatment of FD with radiation therapy,^67^ and thankfully, more conservative approach to FD has become the standard of care.

Radiographically, malignant transformation should be suspected when poorly marginated, mineralized, and osteolytic lesions are identified.^68^ Treatment can include surgery, chemotherapy, and radiation therapy. Transformation often occurs during the fifth decade of life.^66,68^

### Synovial Chondromatosis

**Figure 5:**
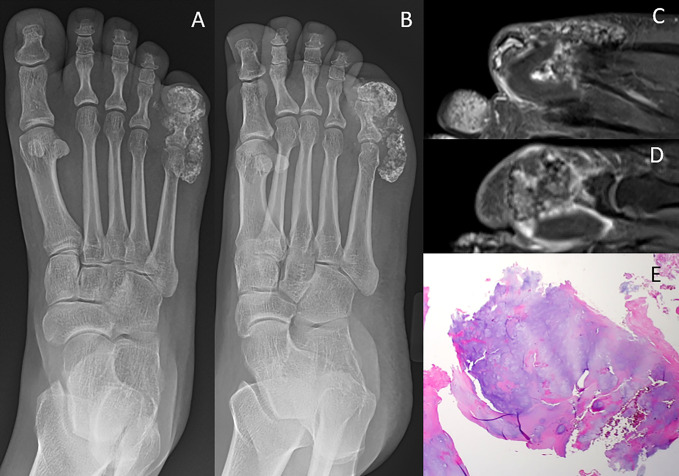
Diagram showing synovial chondromatosis. **A** and **B**, Soft-tissue ossifications around fifth toe. **C** and **D**, Sequential coronal T1 postcontrast MRI with multiple bodies with peripheral enhancement. **E**, Nodules of mature hyaline cartilage are seen with variable degrees of cellularity and nuclear atypia.

The formation of cartilaginous and osteochondral bodies by synovium is the hallmark of synovial chondromatosis. This rare, benign condition can occur in any joint of the body. It often presents nonspecifically and can have a delayed diagnosis of up to 5 years after the onset of symptoms.^69,70^ There is a predilection for weight-bearing joints, with the most common locations being the knee followed by the hip, shoulder, elbow, and ankle.^44^ Overall, it is believed to be a metaplastic process of hyaline cartilaginous with loose body production (Figure 5A–D) that microscopically forms nodules of mature hyaline cartilage with variable cellularity and nuclear atypia (Figure 5E). Typical symptoms include pain, swelling, catching, popping, or crepitus within the affected joint.

Although malignant transformation of synovial chondromatosis is rare, there have been multiple case reports and series.^70,71,72,73,74^ These small sample-sized studies postulate the rate of chondrosarcomatous transformation of up to 6.3%; however, the authors caution that this may be a high estimate because many cases of synovial chondromatosis are asymptomatic.^70^ Typically, low-grade or intermediate-grade chondrosarcoma arises from a range of 2 to 39 years after initial diagnosis, with an average of 20 years.^70^ Clinically differentiating primary synovial chondromatosis from secondary chondrosarcoma can be very difficult,^75^ so clinical suspicion should be raised based on lesion recurrence alone. As is typical of chondrosarcoma treatment, radiation and chemotherapy have limited roles in these patients and surgical intervention is the mainstay of treatment.^76^ Surgical treatment is often wide resection or, if necessary for adequate control, amputation^38^

### Chondroblastoma

Occurring most specifically within the epiphysis of long bones, chondroblastoma is a rare benign primary bone tumor with a frequently aggressive nature.^77,78^ It is most often diagnosed in the second or third decade of life due to pain and often has associated joint symptoms due to its periarticular location. Radiographically, these are seen as well-circumscribed, lytic lesions in the epiphysis. Histologically, there is the proliferation of round chondroblasts in a background of a pink chondroid matrix, interspersed giant cells, and mature cartilage.^77^ Pericellular lace-like calcification is often seen in degenerative chondroblasts. Mutations in the *H3F3B* gene are found in up to 70% of patients with chondroblastoma^79^ and can help differentiate it from other giant-cell containing tumors. Treatment options include radiofrequency ablation in small lesions or local surgical excision with curettage.^80^ Chondroblastoma exhibits a relatively low recurrence rate of approximately 5% to 8%.^81,82^

Although chondroblastoma has metastatic potential itself, with 2% metastasizing to the lung,^83^ more aggressive chondroblastoma may represent its own category of malignant chondroblastoma. However, there is some dispute in malignant chondroblastoma being a separate entity but rather an initial misdiagnosis.^77^ Other malignancies have been found in the setting of chondroblastoma as well, including osteosarcoma and malignant fibrous histiocytoma.^82,83^ Nearly all malignant chondroblastomas occur in patients who have had a previous resection that later develops recurrence of their lesions.^77,82,83^ Prognosis of malignant chondroblastoma is difficult to assess because it is rare and not fully understood; however, metastatic lesions in the setting of benign chondroblastoma portend a poor prognostic implication.^82,84^

### Giant Cell Tumor of Bone

As a benign tumor, giant cell tumor of bone (GCTB) is known to display locally aggressive features with an underrecognized metastatic potential, most often to the lungs.^85,86^ These tumors most often affect the epiphyseal and metaphyseal regions of long bones and are characterized by their classic histologic mononuclear stromal cells with frequent multinucleated giant cells. Bone destruction is mediated through overexpression of RANK ligand, which stimulates precursor monocytes to become the aforementioned osteoclastic giant cells.^87,88^ Mutations in *H3F3A* are present in most GCTB cases, which affects the histone H3.3.^89,90^ The size and overall localized tumor burden of GCTB considerably vary as does the proposed treatment modalities. Systemic adjuvant medical treatment with diphosphonate therapy has been shown to promote apoptosis of the stromal component in GCTB and stabilize inoperable disease.^91,92^ Bisphosphonates may also help prevent local recurrence.^93^ Surgery with extended intralesional curettage, with or without local adjuvant options, is considered the primary treatment modality and the benchmark.^94^ Denosumab, an antibody against receptor activator of nuclear factor-κb-ligand, is a recent treatment option, which has been shown to prevent disease progression in up to 96% of patients in one clinical trial at 13 months.^95^ The overall benefit of denosumab is being called into question, with recent studies showing possible association with malignant transformation^96,97^ and local recurrence in patients undergoing curettage.^98^

Although benign in nature, GCTB does have an ability to metastasize to the lungs, commonly in the setting of recurrent disease or primary axial skeletal location.^99–101^ Lung metastases are often indolent but can be aggressive and fatal.^102^ The metastatic rate in benign tumors is approximately 1% to 9%, although this may not change the long-term outcomes or mortality in these patients.^103,104^ Importantly, the pulmonary metastases are histologically identical to the primary bone lesion.^105,106^ Treatment is usually satisfactory with resection of the pulmonary metastasis.^85,104^

Malignant transformation of GCTB is broken into primary or secondary. Primary malignant GCTB is defined by an area of highly pleomorphic cells within an otherwise benign GCTB, whereas secondary GCTB occurs in an area of previously treated GCTB.^107^ Most malignant GCTBs are secondary to radiation therapy, accounting for up to 75% of all cases.^107^ Comparing malignant versus benign primary GCTB can be very difficult, with only one study finding that benign GCTB was more likely to have well-defined margins and the presence of a thin rim of bone.^108^ Other factors evaluated in the study found that there were no other differences between malignant and benign. Genetic mutations involving *TP53* and *H-RAS* have been identified in secondary malignant GCTB which occur in nonpreviously irradiated patients.^109^ Mortality is influenced by previous radiation therapy, with postirradiation malignancy increasing 5-year mortality from 13% in nonirradiated patients to 72% or greater in postirradiated patients.^107,108^

### Osteoblastoma

Osteoblastoma is a lytic fibro-osseous tumor of bone that produces an osteoid matrix. They were first described as a lesion related to osteoid osteoma, however, with greater growth potential.^110^ Osteoblastoma is differentiated from osteoid osteoma by its larger size (>1.5 cm) and lack of nocturnal night pain relieved by nonsteroidal anti-inflammatory drugs.^111^ However, both entities share similar histology, consisting of trabecular woven bone that is rimmed by plump osteoblasts in a vascularized stroma. As benign neoplasms of bone, osteoblastomas are maybe found incidentally and however are more classically symptomatic.^112^ There is a predilection for the axial spine location with male patients between ages 10 and 25 years being the most common patient cohort.^113–115^ Although benign, these can be locally aggressive with variable clinical course.^112^ These can be differentiated from osteoid osteomas usually by their size, location, and their aggressive nature.^114^ However, both osteoid osteomas and osteoblastomas often carry a *c-FOS* mutation and have other similarities in microscopic morphology.^116^ Treatment is typically with curettage and bone grafting or resection, and prognosis is excellent. There is a 15% to 25% recurrence rate after treatment, typically with curettage and grafting.^117^

Malignant transformation of osteoblastoma to osteosarcoma has been described, most commonly into osteosarcoma after postsurgical resection recurrence.^118,119,120,121^ These case reports however have been called into question as possible initial misdiagnosis due to the similarities in histologic examination.^113^ This counter-argument to true malignant degeneration has been supported by genomic examination.^121^

## Genetic Predisposition

### Retinoblastoma

The tumor suppressor gene retinoblastoma (*RB1*) serves as a cell-cycle checkpoint regulator (Table 3). Lack of the allele, *RB1*, displays Mendelian inheritance patterns in an autosomal dominant fashion. Due to the mutation with subsequent loss of this tumor suppressor gene, various neoplasms can result including osteosarcoma, melanoma, breast, and supratentorial primitive neuroectodermal tumors.^122,123^ Although *RB1* mutation results in classic retinoblastoma of the eye,^122^ osteosarcoma remains the second most common malignancy in this patient cohort.^124^ Screening for retinoblastomas is done in neonates with red reflex testing before discharge from the neonatal nursery.^125^

**Table 3:** Summary of Key Points

No	Genetic Predisposition
1	Retinoblastoma is secondary to loss of tumor suppressor gene *RB1* with classically ocular retinoblastoma formation and often osteosarcoma formation
2	Li-Fraumeni syndrome is characterized by loss of *p53* tumor suppressor gene and lifetime cancer risk greater of 70% to 90%
3	Rothmund-Thompson, Bloom, and Werner syndromes have mutations in genes associated with DNA replication and are at risk for several forms of cancer

Secondary malignancies in patients with retinoblastoma, including osteosarcoma, are common, particularly in the setting of radiation therapy which historically was part of the treatment algorithm.^3,126^ Rates of development are around 13.1% to 38.5% at 30 years or longer after irradiation.^127,128^ However, current rates of secondary malignancy are likely decreasing today because radiation therapy is becoming less frequently used in these patients from 30.5% to 2.6% of cases.^128^ Screening for sarcoma development in patients with heritable retinoblastoma has not been shown to have benefit.^129^

### Li-Fraumeni

Another predisposition syndrome, Li-Fraumeni, has a high association with numerous malignancies. Like *RB1* dysfunction, *TP53* serves as a cell-cycle checkpoint regulator and inherited loss of function mutations displays Mendelian inheritance characteristics in an autosomal dominant fashion.^124^ Heterozygous germline variation in the *TP53* allele results in a lifetime cancer risk of ≥90% for women and ≥70% for men.^130^ The five most common malignancies in these patients are adrenocortical carcinomas, breast cancer, central nervous system tumors, osteosarcomas, and soft-tissue sarcomas.^131^

These patients tend to develop malignancies early in life with 41% occurring before age 18 years,^133^ with osteosarcoma occurring in approximately 12% of individuals.^132^ Families with Li-Fraumeni syndrome do demonstrate anticipation as well, likely secondary to telomerase shortening.^133^ Overall, 3% of osteosarcoma cases are found in Li-Fraumeni patients.^134^ Cancer-screening guidelines for these patients have been described by multiple organizations.^135,136,137,138,139,140^ Screening includes whole-body MRI, laboratory studies, and endoscopy. The Toronto protocol for screening has been shown to have improved overall survival compared with no surveillance.^138^

### Rothmund-Thompson

Because of a mutation of *RECQL4*, a telomerase maintenance protein, Rothmund-Thompson is an autosomal recessive disorder characterized by rash, sparse hair, small size, skeletal and dental abnormalities, and juvenile cataracts.^141,142^ These patients also have an increased risk of cancers, usually osteosarcoma which occurs in 30% to 60% of patients.^133,134,143^ The average age of patients who develop their first malignancy is 15 years, although those who develop osteosarcoma typically do so at an earlier age around 11 years.^143,144^

### Bloom Syndrome

Congenital telangiectatic erythema, or Bloom syndrome, is an autosomal recessive disorder. Genomic instability results from mutations in the *BLM* gene, a RecQ helicase, and patients are predisposed to all types of cancers.^133,145,146^ There is a higher rate of Bloom syndrome in the Ashkenazi Jewish population, accounting for approximately 25% of all cases.^147^ Besides malignancy, these patients often have small stature with proportional bodies, sunlight sensitivities, insulin resistance, and immune abnormalities.^148^

The mean age of death in patients with Bloom syndrome is 26 years, and typically due to complications of malignancy.^145,146^ The most common malignancies in this syndrome are leukemia and lymphoma accounting for 44% of cases with osteosarcoma occurring in approximately 2% of cases.^145^

### Werner Syndrome

Patients with Werner syndrome present with premature aging, bilateral cataracts, short stature, osteoporosis, and hypogonadism.^149^ It is more commonly seen in the Japanese population and is usually caused by mutations in the *WRN* gene, which encodes a RecQ Helicase.^133^ This genetic mutation predisposes these patients to malignancy, often including thyroid neoplasms (16.7% of cases), but also soft-tissue sarcomas (10.1%) and osteosarcomas (7.7%).^150^ When osteosarcoma does develop, it is often in unusual locations such as the foot, ankle, or patella.^133^

## Extrinsic Conditions

### Osteomyelitis

Chronic nonhealing wounds are a well-known risk factor for the development of malignancy, referred to as Marjolin ulcers (Table 4).^151^ They have an incidence of approximately 1.6% to 23% in the setting of chronic osteomyelitis .^152^ Osteomyelitis is the etiology in only 2.6% of Marjolin ulcers however with burns accounting for the vast majority at 76.5%.^153^ The latency period from ulcer development to malignancy is on average 29 to 43 years.^153–155^ The most common location is the lower extremity.^153,155^

**Table 4:** Summary of Key Points

No	Extrinsic Conditions
1	Chronic osteomyelitis with an associated nonhealing wound can result in a Marjolin ulcer or carcinoma formation at the site of the nonhealing wound
2	Postradiation sarcoma is rare but can result in osteosarcoma or soft-tissue sarcomas after doses of 45–60 Gy
3	Extrinsic conditions typically result in secondary malignant transformation 15 + years after radiation or the development of osteomyelitis
4	Chronic osteomyelitis with an associated nonhealing wound can result in a Marjolin ulcer or carcinoma formation at the site of the nonhealing wound

Although squamous cell carcinoma is the most common type of malignancy to develop in this patient population, other malignancies have been identified such as fibrosarcoma, angiosarcoma, osteosarcoma, adenocarcinoma, basal cell carcinoma, and malignant fibrous histiocytoma.^152^ Malignant degeneration may have occurred whether the patient reports worsening pain, increased drainage, enlargement, or lymphadenopathy.^152,154^ Unfortunately, the prognosis in these patients is poor, predominately due to late diagnosis of the malignancy. Metastasis is found in 10% to 27% of patients on initial diagnosis.^153,156^ Treatment has often been with amputation proximal to the tumor,^156–158^ although wide excision with reconstruction may also be a viable option dependent on patient and tumor-specific characteristics.^152^

### Postradiation Sarcoma

Ionizing radiation is a known risk factor for the development of malignancy. Presentation of postradiation sarcoma is on average 15 to 16 years after radiation exposure and most often develops as a bone sarcomas, specifically osteosarcoma.^159–161^ The most common soft-tissue sarcoma to develop is undifferentiated pleomorphic sarcoma.^161,162^ Rates of sarcoma formation after radiation are low at roughly 0.03% to 0.9%.^162,163^ Prior radiation doses of 45 to 60 Gy are often found in these patients, but sarcomas can still arise in lower doses such as 30 Gy.^159,161^ Other risk factors for development are younger age at the time of radiation treatment and concurrent chemotherapy with alkylating agents.^164,165^ Genetic mutations are similar between sporadic and postradiation sarcomas, such as RB1 involvement; however, postradiation sarcomas are more likely to have *CDKN2A* and *CDKN2B*.^166^ The survival rate in these patients is variable in the literature with an average 5-year overall survival of 33% to 68.2%.^159,160,161,162^ Patients presenting without metastatic disease at the time of diagnosis when treated with surgery and chemotherapy may have similar outcomes to primary sarcoma; however, those treated with surgery alone or present with the metastatic disease already present have worse outcomes.^167^

## Conclusion

Multiple factors and conditions that affect bone can predispose patients to the later development of malignancy including benign neoplasms, genetic conditions, and extrinsic factors. Although malignant transformation is rare in many of these conditions, a high index of suspicion must be kept when evaluating and following these patients to provide aggressive appropriate treatment if malignancy develops. Often malignant transformation will present as new-onset pain or mass formation in these patients and should trigger further workup and evaluation for these patients.

## References

[1] Vasquez L Silva J Chavez S : Prognostic impact of diagnostic and treatment delays in children with osteosarcoma. Pediatr Blood Cancer 2020;67:e28180. 31925940 10.1002/pbc.28180

[2] Levine AJ Momand J Finlay CA: The p53 tumour suppressor gene. Nature 1991;351:453-456. 2046748 10.1038/351453a0

[3] Rodriguez-Galindo C Orbach DB VanderVeen D: Retinoblastoma. Pediatr Clin North Am 2015;62:201-223. 25435120 10.1016/j.pcl.2014.09.014

[4] Knudson AG Jr: Mutation and cancer: Statistical study of retinoblastoma. Proc Natl Acad Sci U S A 1971;68:820-823. 5279523 10.1073/pnas.68.4.820 PMC389051

[5] Jhiang SM: The RET proto-oncogene in human cancers. Oncogene 2000;19:5590-5597. 11114739 10.1038/sj.onc.1203857

[6] Kroemer G: The proto-oncogene Bcl-2 and its role in regulating apoptosis. Nat Med 1997;3:614-620. 9176486 10.1038/nm0697-614

[7] Prescott JD Zeiger MA: The RET oncogene in papillary thyroid carcinoma. Cancer 2015;121:2137-2146. 25731779 10.1002/cncr.29044

[8] Kovac M Woolley C Ribi S : Germline RET variants underlie a subset of paediatric osteosarcoma. J Med Genet 2021;58:20-24. 32179705 10.1136/jmedgenet-2019-106734

[9] Coultas L Strasser A: The role of the Bcl-2 protein family in cancer. Semin Cancer Biol 2003;13:115-123. 12654255 10.1016/s1044-579x(02)00129-3

[10] Weinberg RA: Oncogenes and tumor suppressor genes. CA Cancer J Clin 1994;44:160-170. 7621068 10.3322/canjclin.44.3.160

[11] Bozzola M Gertosio C Gnoli M : Hereditary multiple exostoses and solitary osteochondroma associated with growth hormone deficiency: To treat or not to treat? Ital J Pediatr 2015;41:53. 26239617 10.1186/s13052-015-0162-2 PMC4524199

[12] Siegal GP Bloem JL Cates JMM: Soft Tissue and Bone Tumours. Lyon, France, International Agency for Research on Cancer, 2020.

[13] Tong K Liu H Wang X : Osteochondroma: Review of 431 patients from one medical institution in South China. J Bone Oncol 2017;8:23-29. 28932679 10.1016/j.jbo.2017.08.002 PMC5587240

[14] Wicklund CL Pauli RM Johnston D Hecht JT: Natural history study of hereditary multiple exostoses. Am J Med Genet 1995;55:43-46. 7702095 10.1002/ajmg.1320550113

[15] Lamovec J Špiler M Jevtić V: Osteosarcoma arising in a solitary osteochondroma of the fibula. Arch Pathol Lab Med 1999;123:832-834. 10458834 10.5858/1999-123-0832-OAIASO

[16] Florez B Monckeberg J Castillo G Beguiristain J: Solitary osteochondroma long-term follow-up. J Pediatr Orthop 2008;17:91-94. 10.1097/bpb.0b013e3282f450c3 18510166

[17] Ahmed AR Tan TS Unni KK Collins MS Wenger DE Sim FH: Secondary chondrosarcoma in osteochondroma: Report of 107 patients. Clin Orthop Relat Res 2003:193-206. 10.1097/01.blo.0000069888.31220.2b 12782876

[18] Pedrini E Jennes I Tremosini M : Genotype-phenotype correlation study in 529 patients with multiple hereditary exostoses: Identification of “protective” and “risk” factors. J Bone Joint Surg Am 2011;93:2294-2302. 22258776 10.2106/JBJS.J.00949

[19] Fei L Ngoh C Porter DE: Chondrosarcoma transformation in hereditary multiple exostoses: A systematic review and clinical and cost-effectiveness of a proposed screening model. J Bone Oncol 2018;13:114-122. 30591865 10.1016/j.jbo.2018.09.011 PMC6303411

[20] Jurik AG Jørgensen PH Mortensen MM: Whole-body MRI in assessing malignant transformation in multiple hereditary exostoses and enchondromatosis: Audit results and literature review. Skeletal Radiol 2020;49:115-124. 31273432 10.1007/s00256-019-03268-z

[21] Legeai-Mallet L Munnich A Maroteaux P Le Merrer M Munnich A: Incomplete penetrance and expressivity skewing in hereditary multiple exostoses. Clin Genet 1997;52:12-16. 9272707 10.1111/j.1399-0004.1997.tb02508.x

[22] Jurik AG: Multiple hereditary exostoses and enchondromatosis. Best Pract Res Clin Rheumatol 2020;34:101505. 32253147 10.1016/j.berh.2020.101505

[23] Beltrami G Ristori G Scoccianti G Tamburini A Capanna R: Hereditary multiple exostoses: A review of clinical appearance and metabolic pattern. Clin Cases Miner Bone Metab 2016;13:110-118. 27920806 10.11138/ccmbm/2016.13.2.110 PMC5119707

[24] Stieber JR Dormans JP: Manifestations of hereditary multiple exostoses. J Am Acad Orthop Surg 2005;13:110-120. 15850368 10.5435/00124635-200503000-00004

[25] Jennes I Pedrini E Zuntini M : Multiple osteochondromas: Mutation update and description of the multiple osteochondromas mutation database (MOdb). Hum Mutat 2009;30:1620-1627. 19810120 10.1002/humu.21123

[26] Wells M Birchard Z: A 40-year-old male presenting with hereditary multiple exostosis: Management and considerations. Case Rep Orthop 2019;2019:4793043-4793044. 31001440 10.1155/2019/4793043 PMC6436337

[27] Lin PP Moussallem CD Deavers MT: Secondary chondrosarcoma. J Am Acad Orthop Surg 2010;18:608-615. 20889950 10.5435/00124635-201010000-00004

[28] Bernard SA Murphey MD Flemming DJ Kransdorf MJ: Improved differentiation of benign osteochondromas from secondary chondrosarcomas with standardized measurement of cartilage cap at CT and MR imaging. Radiology 2010;255:857-865. 20392983 10.1148/radiol.10082120

[29] Altay M Bayrakci K Yildiz Y Erekul S Saglik Y: Secondary chondrosarcoma in cartilage bone tumors: Report of 32 patients. J Orthop Sci 2007;12:415-423. 17909925 10.1007/s00776-007-1152-z

[30] Bus MPA Campanacci DA Albergo JI : Conventional primary central chondrosarcoma of the pelvis: Prognostic factors and outcome of surgical treatment in 162 patients. J Bone Joint Surg Am 2018;100:316-325. 29462035 10.2106/JBJS.17.00105

[31] Herget GW Strohm P Rottenburger C : Insights into Enchondroma, Enchondromatosis and the risk of secondary Chondrosarcoma. Review of the literature with an emphasis on the clinical behaviour, radiology, malignant transformation and the follow up. Neoplasma 2014;61:365-378. 24645839 10.4149/neo_2014_046

[32] Mulligan ME: How to diagnose enchondroma, bone infarct, and chondrosarcoma. Curr Probl Diagn Radiol 2019;48:262-273. 29724496 10.1067/j.cpradiol.2018.04.002

[33] Adler C-P: Bone Diseases: Macroscopic, Histological, and Radiological Diagnosis of Structural Changes in the Skeleton, Freiburg, Germany, Springer, 2000, Vol. 588.

[34] Sassoon AA Fitz-Gibbon PD Harmsen WS Moran SL: Enchondromas of the hand: Factors affecting recurrence, healing, motion, and malignant transformation. J Hand Surg Am 2012;37:1229-1234. 22542061 10.1016/j.jhsa.2012.03.019

[35] Verdegaal SHM Bovée JVMG Pansuriya TC : Incidence, predictive factors, and prognosis of chondrosarcoma in patients with Ollier disease and Maffucci syndrome: An International Multicenter Study of 161 patients. Oncologist 2011;16:1771-1779. 22147000 10.1634/theoncologist.2011-0200 PMC3248776

[36] Amary MF Damato S Halai D : Ollier disease and Maffucci syndrome are caused by somatic mosaic mutations of IDH1 and IDH2. Nat Genet 2011;43:1262-1265. 22057236 10.1038/ng.994

[37] Pansuriya TC Van Eijk R D'Adamo P : Somatic mosaic IDH1 and IDH2 mutations are associated with enchondroma and spindle cell hemangioma in Ollier disease and Maffucci syndrome. Nat Genet 2011;43:1256-1261. 22057234 10.1038/ng.1004 PMC3427908

[38] Wells ME Eckhoff MD Kafchinski LA Polfer EM Potter BK: Conventional cartilaginous tumors: Evaluation and treatment. JBJS Rev 2021;9. 10.2106/JBJS.RVW.20.00159 34881859

[39] El Abiad JM Robbins SM Cohen B : Natural history of Ollier disease and Maffucci syndrome: Patient survey and review of clinical literature. Am J Med Genet 2020;182:1093-1103. 32144835 10.1002/ajmg.a.61530 PMC8164175

[40] Amary MF Bacsi K Maggiani F : IDH1 and IDH2 mutations are frequent events in central chondrosarcoma and central and periosteal chondromas but not in other mesenchymal tumours. J Pathol 2011;224:334-343. 21598255 10.1002/path.2913

[41] Weinschenk RC Wang WL Lewis VO: Chondrosarcoma. J Am Acad Orthop Surg 2021;29:553-562. 33595238 10.5435/JAAOS-D-20-01188

[42] Lubahn JD Bachoura A: Enchondroma of the hand: Evaluation and management. J Am Acad Orthop Surg 2016;24:625-633. 27454024 10.5435/JAAOS-D-15-00452

[43] Bus MPA Campanacci DA Albergo JI : Conventional primary central chondrosarcoma of the pelvis: Prognostic factors and outcome of surgical treatment in 162 patients. J Bone Joint Surg Am 2018;100:316-325. 29462035 10.2106/JBJS.17.00105

[44] Biermann JS Siegel GW, American Academy of Orthopaedic Surgeons: Orthopaedic knowledge update Musculoskeletal tumors. Rosemont, IL, American Academy of Orthopaedic Surgeons, pp 528.

[45] Van Staa TP Selby P Leufkens HGM Lyles K Sprafka JM Cooper C: Incidence and natural history of Paget's disease of bone in England and Wales. J Bone Miner Res 2002;17:465-471. 11878305 10.1359/jbmr.2002.17.3.465

[46] Ralston SH: Clinical practice. Paget's disease of bone. N Engl J Med 2013;368:644, 650. 23406029 10.1056/NEJMcp1204713

[47] Layfield R: The molecular pathogenesis of Paget disease of bone. Expert Rev Mol Med 2007;9:1-13. 10.1017/S1462399407000464 17903332

[48] Smith SE Murphey MD Motamedi K Mulligan ME Resnik CS Gannon FH: From the archives of the AFIP: Radiologic spectrum of paget disease of bone and its complications with pathologic correlation. Radiographics 2002;22:1191-1216. 12235348 10.1148/radiographics.22.5.g02se281191

[49] Harvey L Gray T Beneton MNC Douglas DL Kanis JA Russell RGG: Ultrastructural features of the osteoclasts from Paget's disease of bone in relation to a viral aetiology. J Clin Pathol 1982;35:771-779. 7096600 10.1136/jcp.35.7.771 PMC497776

[50] Schajowicz F Santini Araujo E Berenstein M: Sarcoma complicating Paget's disease of bone. A clinicopathological study of 62 cases. J Bone Joint Surg Br 1983;65:299-307. 6573330 10.1302/0301-620X.65B3.6573330

[51] Shaylor PJ Peake D Grimer RJ Carter SR Tillman RM Spooner D: Paget's osteosarcoma - No cure in sight. Sarcoma 1999;3:191-192. 18521284 10.1080/13577149977631 PMC2395430

[52] Frassica FJ Sim FH Frassica DA Wold LE: Survival and management considerations in postirradiation osteosarcoma and Paget's osteosarcoma. Clin Orthop Relat Res 1991:120-127. 1884530

[53] Dray MS Miller MV: Paget's osteosarcoma and post-radiation osteosarcoma: Secondary osteosarcoma at Middlemore Hospital, New Zealand. Pathology 2008;40:604-610. 18752128 10.1080/00313020802320663

[54] Calabrò T Mavrogenis AF Ruggieri P: Osteoblastic osteosarcoma in monostotic Paget's disease. Musculoskelet Surg 2011;95:37-40. 21409504 10.1007/s12306-011-0100-4

[55] López C Thomas DV Davies AM: Neoplastic transformation and tumour-like lesions in Paget's disease of bone: A pictorial review. Eur Radiol 2003;13(Suppl 4):L151-L163. 15018182 10.1007/s00330-003-1927-3

[56] Colina M La Corte R De Leonardis F Trotta F: Paget's disease of bone: A review. Rheumatol Int 2008;28:1069-1075. 18592244 10.1007/s00296-008-0640-6

[57] Seitz S Priemel M Zustin J : Paget's disease of bone: Histologic analysis of 754 patients. J Bone Miner Res 2009;24:62-69. 18767930 10.1359/jbmr.080907

[58] Mangham DC Davie MW Grimer RJ: Sarcoma arising in Paget's disease of bone: Declining incidence and increasing age at presentation. Bone 2009;44:431-436. 19064007 10.1016/j.bone.2008.11.002

[59] Mirabello L Troisi RJ Savage SA: Osteosarcoma incidence and survival rates from 1973 to 2004: Data from the surveillance, epidemiology, and end results program. Cancer 2009;115:1531-1543. 19197972 10.1002/cncr.24121 PMC2813207

[60] Mankin HJ Hornicek FJ: Paget's sarcoma: A historical and outcome review. Clin Orthop Relat Res 2005;438:97-102. 16131876 10.1097/01.blo.0000180053.99840.27

[61] Leet AI Collins MT: Current approach to fibrous dysplasia of bone and McCune–Albright syndrome. J Child Orthop 2007;1:3-17. 19308500 10.1007/s11832-007-0006-8 PMC2656698

[62] Riddle ND Bui MM: Fibrous dysplasia. Arch Pathol Lab Med 2013;137:134-138. 23276185 10.5858/arpa.2012.0013-RS

[63] Most MJ Sim FH Inwards CY: Osteofibrous dysplasia and adamantinoma. J Am Acad Orthop Surg 2010;18:358-366. 20511441 10.5435/00124635-201006000-00008

[64] Parekh SG Donthineni-Rao R Ricchetti E Lackman RD: Fibrous dysplasia. J Am Acad Orthop Surg 2004;12:305-313. 15469225 10.5435/00124635-200409000-00005

[65] Boyce AM Florenzano P de Castro LF Collins MT: Fibrous Dysplasia/McCune-Albright Syndrome. Leiden, the Netherlands, Leiden University, 2019.

[66] Ruggieri P Sim FH Bond JR Unni KK: Malignancies in fibrous dysplasia. Cancer 1994;73:1411-1424. 8111708 10.1002/1097-0142(19940301)73:5<1411::aid-cncr2820730516>3.0.co;2-t

[67] Stanton RP Ippolito E Springfield D Lindaman L Wientroub S Leet A: The surgical management of fibrous dysplasia of bone. Orphanet J Rare Dis 2012;7(suppl 1):1-9. 22640754 10.1186/1750-1172-7-S1-S1 PMC3359959

[68] Qu N Yao W Cui X Zhang H: Malignant transformation in monostotic fibrous dysplasia: Clinical features, imaging features, outcomes in 10 patients, and review. Medicine (Baltimore) 2015;94:e369. 25621678 10.1097/MD.0000000000000369 PMC4602648

[69] Neumann JA Garrigues GE Brigman BE Eward WC: Synovial chondromatosis. JBJS Rev 2016;4:e2. 10.2106/JBJS.RVW.O.00054 27490219

[70] Evans S Boffano M Chaudhry S Jeys L Grimer R: Synovial chondrosarcoma arising in synovial chondromatosis. Sarcoma 2014;2014:647939. 24737946 10.1155/2014/647939 PMC3967817

[71] Kenan S Abdelwahab IF Klein MJ Lewis MM: Case report 817: Synovial chondrosarcoma secondary to synovial chondromatosis. Skeletal Radiol 1993;22:623-626. 8291018 10.1007/BF00197149

[72] Sachinis NP Sinopidis C Baliaka A Givissis P: Odyssey of an elbow synovial chondromatosis. Orthopedics 2015;38:e62-e67. 25611422 10.3928/01477447-20150105-91

[73] Davis RI HAmilton A Biggart JD HAmilton A: Primary synovial chondromatosis: A clinicopathologic review and assessment of malignant potential. Hum Pathol 1998;29:683-688. 9670824 10.1016/s0046-8177(98)90276-3

[74] Bhadra AK Pollock R Tirabosco RP : Primary tumours of the synovium: A report of four cases of malignant tumour. J Bone Joint Surg Br 2007;89:1504-1508. 17998190 10.1302/0301-620X.89B11.18963

[75] Murphey MD Vidal JA Fanburg-Smith JC Gajewski DA: From the archives of the AFIP: Imaging of synovial chondromatosis with radiologic-pathologic correlation. Radiographics 2007;27:1465-1488. 17848703 10.1148/rg.275075116

[76] Gelderblom H Hogendoorn PCW Dijkstra SD : The clinical approach towards chondrosarcoma. Oncologist 2008;13:320-329. 18378543 10.1634/theoncologist.2007-0237

[77] Chen W DiFrancesco LM: Chondroblastoma: An update. Arch Pathol Lab Med 2017;141:867-871. 28557595 10.5858/arpa.2016-0281-RS

[78] Ramappa AJ Lee FY Tang P Carlson JR Gebhardt MC Mankin HJ: Chondroblastoma of bone. J Bone Joint Surg Am 2000;82:1140-1145. 10954104

[79] Cleven AHG Höcker S Briaire-De Bruijn I Szuhai K Cleton-Jansen AM Bovée JVMG: Mutation analysis of H3F3A and H3F3B as a diagnostic tool for giant cell tumor of bone and chondroblastoma. Am J Surg Pathol 2015;39:1576-1583. 26457357 10.1097/PAS.0000000000000512

[80] Rybak LD Rosenthal DI Wittig JC: Chondroblastoma: Radiofrequency ablation--alternative to surgical resection in selected cases. Radiology 2009;251:599-604. 19304917 10.1148/radiol.2512080500

[81] Xu H Nugent D Monforte HL : Chondroblastoma of bone in the extremities: A multicenter retrospective study. J Bone Joint Surg Am 2015;97:925-931. 26041854 10.2106/JBJS.N.00992

[82] Lin PP Thenappan A Deavers MT Lewis VO Yasko AW: Treatment and prognosis of chondroblastoma. Clin Orthop Relat Res 2005;438:103-109. 16131877 10.1097/01.blo.0000179591.72844.c3

[83] Narhari MD Haseeb A Lee S Singh V: Spontaneous conventional osteosarcoma transformation of a chondroblastoma: A case report and literature review. Indian J Orthop 2018;52:87-90. 29416176 10.4103/ortho.IJOrtho_495_17 PMC5791238

[84] Laitinen MK Stevenson JD Evans S : Chondroblastoma in pelvis and extremities- A single centre study of 177 cases. J Bone Oncol 2019;17:100248. 31428555 10.1016/j.jbo.2019.100248 PMC6695276

[85] Raskin KA Schwab JH Mankin HJ Springfield DS Hornicek FJ: Giant cell tumor of bone. J Am Acad Orthop Surg 2013;21:118-126. 23378375 10.5435/JAAOS-21-02-118

[86] Sobti A Agrawal P Agarwala S Agarwal M: Giant cell tumor of bone - An overview. Arch Bone Joint Surg 2016;4:2-9. 26894211 PMC4733230

[87] Kim Y Nizami S Goto H Lee FY: Modern interpretation of giant cell tumor of bone: Predominantly osteoclastogenic stromal tumor. Clin Orthop Surg 2012;4:107-116. 22662295 10.4055/cios.2012.4.2.107 PMC3360182

[88] Wu PF Tang JY Li KH: RANK pathway in giant cell tumor of bone: Pathogenesis and therapeutic aspects. Tumour Biol 2015;36:495-501. 25618600 10.1007/s13277-015-3094-y

[89] Yamamoto H Ishihara S Toda Y Oda Y: Histone H3.3 mutation in giant cell tumor of bone: An update in pathology. Med Mol Morphol 2020;53:1-6. 31748824 10.1007/s00795-019-00238-1

[90] Behjati S Tarpey PS Presneau N : Distinct H3F3A and H3F3B driver mutations define chondroblastoma and giant cell tumor of bone. Nat Genet 2013;45:1479-1482. 24162739 10.1038/ng.2814 PMC3839851

[91] Chang SS Suratwala SJ Jung KM : Bisphosphonates may reduce recurrence in giant cell tumor by inducing apoptosis. Clin Orthop Relat Res 2004;426:103-109. 10.1097/01.blo.0000141372.54456.80 15346059

[92] Balke M Campanacci L Gebert C : Bisphosphonate treatment of aggressive primary, recurrent and metastatic giant cell tumour of bone. BMC Cancer 2010;10:462. 20799989 10.1186/1471-2407-10-462 PMC2940802

[93] Tse LF Wong KC Kumta SM Huang L Chow TC Griffith JF: Bisphosphonates reduce local recurrence in extremity giant cell tumor of bone: A case–control study. Bone 2008;42:68-73. 17962092 10.1016/j.bone.2007.08.038

[94] Network NCC. Bone Cancer 2022. https://www.nccn.org/professionals/physician_gls/pdf/bone.pdf.

[95] Chawla S Henshaw R Seeger L : Safety and efficacy of denosumab for adults and skeletally mature adolescents with giant cell tumour of bone: Interim analysis of an open-label, parallel-group, phase 2 study. Lancet Oncol 2013;14:901-908. 23867211 10.1016/S1470-2045(13)70277-8

[96] Park A Cipriano CA Hill K Kyriakos M McDonald DJ: Malignant transformation of a giant cell tumor of bone treated with denosumab: A case report. JBJS Case Connect 2016;6:e78. 29252655 10.2106/JBJS.CC.16.00024

[97] Hasenfratz M Mellert K Marienfeld R : Profiling of three H3F3A-mutated and denosumab-treated giant cell tumors of bone points to diverging pathways during progression and malignant transformation. Sci Rep 2021;11:5709. 33707617 10.1038/s41598-021-85319-x PMC7952552

[98] Asano N Saito M Kobayashi E : Preoperative denosumab therapy against giant cell tumor of bone is associated with an increased risk of local recurrence after curettage surgery. Ann Surg Oncol 2022;29:3992-4000, doi:35175454 10.1245/s10434-022-11411-9

[99] Niu X Zhang Q Hao L : Giant cell tumor of the extremity: Retrospective analysis of 621 Chinese patients from one institution. J Bone Joint Surg Am 2012;94:461-467. 22398741 10.2106/JBJS.J.01922

[100] Kremen TJ Bernthal NM Eckardt MA Eckardt JJ: Giant cell tumor of bone: Are we stratifying results appropriately?. Clin Orthop Relat Res 2012;470:677-683. 22125240 10.1007/s11999-011-2172-8 PMC3270191

[101] Chan CM Adler Z Reith JD Gibbs CP: Risk factors for pulmonary metastases from giant cell tumor of bone. J Bone Joint Surg Am 2015;97:420-428. 25740033 10.2106/JBJS.N.00678

[102] Balke M Schremper L Gebert C : Giant cell tumor of bone: Treatment and outcome of 214 cases. J Cancer Res Clin Oncol 2008;134:969-978. 18322700 10.1007/s00432-008-0370-x PMC12160765

[103] Rosario M Kim HS Yun JY Han I: Surveillance for lung metastasis from giant cell tumor of bone. J Surg Oncol 2017;116:907-913. 28650536 10.1002/jso.24739

[104] Viswanathan S Jambhekar NA: Metastatic giant cell tumor of bone: Are there associated factors and best treatment modalities? Clin Orthop Relat Res 2010;468:827-833. 19597900 10.1007/s11999-009-0966-8 PMC2816751

[105] Kay RM Eckardt JJ Seeger LL Mirra JM Hak DJ: Pulmonary metastasis of benign giant cell tumor of bone. Six histologically confirmed cases, including one of spontaneous regression. Clin Orthop Relat Res 1994:219-230. 8168305

[106] Tubbs WS Brown LR Beabout JW Rock MG Unni KK: Benign giant-cell tumor of bone with pulmonary metastases: Clinical findings and radiologic appearance of metastases in 13 cases. AJR Am J Roentgenol 1992;158:331-334. 1729794 10.2214/ajr.158.2.1729794

[107] Palmerini E Picci P Reichardt P Downey G: Malignancy in giant cell tumor of bone: A review of the literature. Technol Cancer Res Treat 2019;18:1533033819840000. 30935298 10.1177/1533033819840000 PMC6446439

[108] Domovitov SV Healey JH: Primary malignant giant-cell tumor of bone has high survival rate. Ann Surg Oncol 2010;17:694-701. 19902306 10.1245/s10434-009-0803-z

[109] Oda Y SAkAmoto A SaiTo T : Secondary malignant giant-cell tumour of bone: Molecular abnormalities of p53 and H-ras gene correlated with malignant transformation. Histopathology 2001;39:629-637. 11903582 10.1046/j.1365-2559.2001.01275.x

[110] Lichtenstein L Sawyer WR: Benign Osteoblastoma. Further observations and report of twenty additional cases. J Bone Joint Surg Am 1964;46:755-765. 14161088

[111] Gitelis S Schajowicz F: Osteoid osteoma and osteoblastoma. Orthop Clin North Am 1989;20:313-325. 2662110

[112] Yalcinkaya U Doganavsargil B Sezak M : Clinical and morphological characteristics of osteoid osteoma and osteoblastoma: A retrospective single-center analysis of 204 patients. Ann Diagn Pathol 2014;18:319-325. 25224389 10.1016/j.anndiagpath.2014.08.006

[113] Limaiem F Byerly DW Singh R, Osteoblastoma. StatPearls, 2021. https://www.ncbi.nlm.nih.gov/books/NBK536954/. 30725639

[114] Atesok KI Alman BA Schemitsch EH Peyser A Mankin H: Osteoid osteoma and osteoblastoma. J Am Acad Orthop Surg 2011;19:678-689. 22052644 10.5435/00124635-201111000-00004

[115] Arkader A Dormans JP: Osteoblastoma in the skeletally immature. J Pediatr Orthop 2008;28:555-560. 18580372 10.1097/BPO.0b013e31817bb849

[116] Amary F Flanagan AM O'Donnell P: Benign bone-forming tumors. Surg Pathol Clin 2021;14:549-565. 34742480 10.1016/j.path.2021.06.002

[117] Berry M Mankin H Gebhardt M Rosenberg A Hornicek F: Osteoblastoma: A 30-year study of 99 cases. J Surg Oncol 2008;98:179-183. 18561158 10.1002/jso.21105

[118] Lucas DR Unni KK McLeod RA O'Connor MI Sim FH: Osteoblastoma: Clinicopathologic study of 306 cases. Hum Pathol 1994;25:117-134. 8119712 10.1016/0046-8177(94)90267-4

[119] Mayer L: Malignant degeneration of so-called benign osteoblastoma. Bull Hosp Joint Dis 1967;28:4-13. 5232357

[120] Görgün O Salduz A Kebudi R Özger H Bilgiç B: Malignant transformation of aggressive osteoblastoma to osteosarcoma. Eklem Hastalik Cerrahisi 2016;27:108-112. 27499324 10.5606/ehc.2016.23

[121] Geller DS Levine NL Hoang BH : Genomic analysis does not support malignant transformation of osteoblastoma to osteosarcoma. JCO Precis Oncol 2019;3:1-7. 10.1200/PO.19.00166 PMC7446473 32914027

[122] Dimaras H Kimani K Dimba EAO : Retinoblastoma. Lancet 2012;379:1436-1446. 22414599 10.1016/S0140-6736(11)61137-9

[123] Kleinerman RA Tucker MA Tarone RE : Risk of new cancers after radiotherapy in long-term survivors of retinoblastoma: An extended follow-up. J Clin Oncol 2005;23:2272-2279. 15800318 10.1200/JCO.2005.05.054

[124] Ito M Barys L O'Reilly T : Comprehensive mapping of p53 pathway alterations reveals an apparent role for both SNP309 and MDM2 amplification in sarcomagenesis. Clin Cancer Res 2011;17:416-426. 21159888 10.1158/1078-0432.CCR-10-2050

[125] Retinoblastoma. Am Acad Pediatr, 2020. https://aapos.org/glossary/retinoblastoma.

[126] Wong FL Boice JD Abramson DH : Cancer incidence after retinoblastoma. Radiation dose and sarcoma risk. JAMA 1997;278:1262-1267. 9333268 10.1001/jama.278.15.1262

[127] Kleinerman RA Tucker MA Abramson DH Seddon JM Tarone RE Fraumeni JF: Risk of soft tissue sarcomas by individual subtype in survivors of hereditary retinoblastoma. J Natl Cancer Inst 2007;99:24-31. 17202110 10.1093/jnci/djk002

[128] Shinohara ET DeWees T Perkins SM: Subsequent malignancies and their effect on survival in patients with retinoblastoma. Pediatr Blood Cancer 2014;61:116-119. 23918737 10.1002/pbc.24714

[129] Tonorezos ES Friedman DN Barnea D : Recommendations for long-term follow-up of adults with heritable retinoblastoma. Ophthalmology 2020;127:1549-1557. 32422154 10.1016/j.ophtha.2020.05.024 PMC7606265

[130] Schneider K Zelley K Nichols KE Garber J, Li-Fraumeni Syndrome. GeneReviews(®), 2019. http://europepmc.org/books/NBK1311. 20301488

[131] Bougeard G Renaux-Petel M Flaman JM : Revisiting Li-Fraumeni syndrome from TP53 mutation carriers. J Clin Oncol 2015;33:2345-2352. 26014290 10.1200/JCO.2014.59.5728

[132] Mirabello L Yeager M Mai PL : Germline TP53 variants and susceptibility to osteosarcoma. J Natl Cancer Inst 2015;107:101. 10.1093/jnci/djv101 PMC4651039 25896519

[133] Hameed M Mandelker D: Tumor syndromes predisposing to osteosarcoma. Adv Anat Pathol 2018;25:217-222. 29668499 10.1097/PAP.0000000000000190 PMC6688172

[134] Ottaviani G Jaffe N: The epidemiology of osteosarcoma. Cancer Treat Res 2009;152:3-13. 20213383 10.1007/978-1-4419-0284-9_1

[135] Ballinger ML Mitchell G Thomas DM: Surveillance recommendations for patients with germline TP53 mutations. Curr Opin Oncol 2015;27:332-337. 26049273 10.1097/CCO.0000000000000200

[136] McBride KA Ballinger ML Killick E : Li-Fraumeni syndrome: Cancer risk assessment and clinical management. Nat Rev Clin Oncol 2014;11:260-271. 24642672 10.1038/nrclinonc.2014.41

[137] Daly MB Pilarski R Axilbund JE : NCCN clinical practical guidelines in oncology genetic/familial high-risk assessment: Breast and ovarian. Natl Compr Cancer Netw 2017. https://www.nccn.org/professionals/physician_gls/f_guidelines.asp#genetics_screening.

[138] Villani A Shore A Wasserman JD : Biochemical and imaging surveillance in germline TP53 mutation carriers with Li-Fraumeni syndrome: 11 year follow-up of a prospective observational study. Lancet Oncol 2016;17:1295-1305. 27501770 10.1016/S1470-2045(16)30249-2

[139] Villani A Tabori U Schiffman J : Biochemical and imaging surveillance in germline TP53 mutation carriers with Li-Fraumeni syndrome: A prospective observational study. Lancet Oncol 2011;12:559-567. 21601526 10.1016/S1470-2045(11)70119-X

[140] Kratz CP Achatz MI Brugieres L : Cancer screening recommendations for individuals with Li-Fraumeni syndrome. Clin Cancer Res 2017;23:e38-e45. 28572266 10.1158/1078-0432.CCR-17-0408

[141] Wang LL Plon SE, Rothmund-Thomson Syndrome. GeneReviews®. 2020. https://www.ncbi.nlm.nih.gov/books/NBK1237/.

[142] Ghosh AK Rossi ML Singh DK : RECQL4, the protein mutated in Rothmund-Thomson syndrome, functions in telomere maintenance. J Biol Chem 2012;287:196-209. 22039056 10.1074/jbc.M111.295063 PMC3249070

[143] Wang LL Gannavarapu A Kozinetz CA : Association between osteosarcoma and deleterious mutations in the RECQL4 gene in Rothmund-Thomson syndrome. J Natl Cancer Inst 2003;95:669-674. 12734318 10.1093/jnci/95.9.669

[144] Simon T Kohlhase J Wilhelm C Kochanek M De Carolis B Berthold F: Multiple malignant diseases in a patient with Rothmund–Thomson syndrome with RECQL4 mutations: Case report and literature review. Am J Med Genet 2010;152A:1575-1579. 20503338 10.1002/ajmg.a.33427

[145] German J: Bloom's syndrome. XX. The first 100 cancers. Cancer Genet Cytogenet 1997;93:100-106. 9062585 10.1016/s0165-4608(96)00336-6

[146] Hafsi W Badri T Rice AS. Bloom Syndrome. StatPearls. 2021. https://www.ncbi.nlm.nih.gov/books/NBK448138/. 28846287

[147] Li L Eng C Desnick RJ German J Ellis NA: Carrier frequency of the Bloom syndrome blmAsh mutation in the Ashkenazi Jewish population. Mol Genet Metab 1998;64:286-290. 9758720 10.1006/mgme.1998.2733

[148] Flanagan M Cunniff C. Bloom Syndrome. NCBI Bookshelf. 2019. http://europepmc.org/books/NBK1398.

[149] Oshima J Sidorova JM Monnat RJ: Werner syndrome: Clinical features, pathogenesis and potential therapeutic interventions. Ageing Res Rev 2017;33:105-114. 26993153 10.1016/j.arr.2016.03.002 PMC5025328

[150] Lauper JM Krause A Vaughan TL Monnat RJ: Spectrum and risk of neoplasia in werner syndrome: A systematic review. PLoS One 2013;8:e59709. 23573208 10.1371/journal.pone.0059709 PMC3613408

[151] Multhoff G Molls M Radons J: Chronic inflammation in cancer development. Front Immunol 2011;2:98. 22566887 10.3389/fimmu.2011.00098 PMC3342348

[152] Panteli M Puttaswamaiah R Lowenberg DW Giannoudis PV: Malignant transformation in chronic osteomyelitis: Recognition and principles of management. J Am Acad Orthop Surg 2014;22:586-594. 25157040 10.5435/JAAOS-22-09-586

[153] Kerr-Valentic MA Samimi K Rohlen BH Agarwal JP Rockwell WB: Marjolin's ulcer: Modern analysis of an ancient problem. Plast Reconstr Surg 2009;123:184-191. 19116552 10.1097/PRS.0b013e3181904d86

[154] Bauer T David T Rimareix F Lortat-Jacob A LortAt-JAcob A: Marjolin's ulcer in chronic osteomyelitis: Seven cases and a review of the literature [in French]. Rev Chir Orthop Reparatrice Appar Mot 2007;93:63-71. 17389826 10.1016/s0035-1040(07)90205-6

[155] Onah II Olaitan PB Ogbonnaya IS Onuigbo WIB: Marjolin's ulcer (correction of ulcer) at a Nigerian hospital (1993-2003). J Plast Reconstr Aesthet Surg 2006;59:565-566. 16631566 10.1016/j.bjps.2005.11.003

[156] Altay M Arikan M Yildiz Y Saglik Y: Squamous cell carcinoma arising in chronic osteomyelitis in foot and ankle. Foot Ankle Int 2004;25:805-809. 15574240 10.1177/107110070402501109

[157] Alami M Mahfoud M El Bardouni A Berrada MS El Yaacoubi M: Squamous cell carcinoma arising from chronic osteomyelitis. Acta Orthop Traumatol Turc 2011;45:144-148. 21765226 10.3944/AOTT.2011.2537

[158] Pandey M Kumar P Khanna AK: Marjolin's ulcer associated with chronic osteomyelitis. J Wound Care 2009;18:504-506. 20081575 10.12968/jowc.2009.18.12.45607

[159] Inoue YZ Frassica FJ Sim FH Unni KK Petersen IA McLeod RA: Clinicopathologic features and treatment of postirradiation sarcoma of bone and soft tissue. J Surg Oncol 2000;75:42-50. 11025461 10.1002/1096-9098(200009)75:1<42::aid-jso8>3.0.co;2-g

[160] Mavrogenis AF Pala E Guerra G Ruggieri P: Post-radiation sarcomas. Clinical outcome of 52 Patients. J Surg Oncol 2012;105:570-576. 22012601 10.1002/jso.22122

[161] Joo MW Kang YK Ogura K : Post-radiation sarcoma: A study by the Eastern Asian Musculoskeletal Oncology Group. PLoS One 2018;13:e0204927. 30332455 10.1371/journal.pone.0204927 PMC6192585

[162] Bjerkehagen B Smeland S Walberg L : Radiation-induced sarcoma: 25-year experience from the Norwegian radium hospital. Acta Oncologica 2008;47:1475-1482. 18607853 10.1080/02841860802047387

[163] Kim KS Chang JH Choi N : Radiation-induced sarcoma: A 15-year experience in a single large tertiary referral center. Cancer Res Treat 2016;48:650-657. 27004955 10.4143/crt.2015.171 PMC4843709

[164] Virtanen A Pukkala E Auvinen A: Incidence of bone and soft tissue sarcoma after radiotherapy: A cohort study of 295, 712 Finnish cancer patients. Int J Cancer 2006;118:1017-1021. 16152578 10.1002/ijc.21456

[165] Menu-Branthomme A Rubino C Shamsaldin A : Radiation dose, chemotherapy and risk of soft tissue sarcoma after solid tumours during childhood. Int J Cancer 2004;110:87-93. 15054872 10.1002/ijc.20002

[166] Lesluyes T Baud J Pérot G : Genomic and transcriptomic comparison of post-radiation versus sporadic sarcomas. Mod Pathol 2019;32:1786-1794. 31243333 10.1038/s41379-019-0300-2

[167] Shaheen M Deheshi BM Riad S : Prognosis of radiation-induced bone sarcoma is similar to primary osteosarcoma. Clin Orthop Relat Res 2006;450:76-81. 16906097 10.1097/01.blo.0000229315.58878.c1

